# Identification and analysis of OsttaDSP, a phosphoglucan phosphatase from *Ostreococcus tauri*

**DOI:** 10.1371/journal.pone.0191621

**Published:** 2018-01-23

**Authors:** Julieta B. Carrillo, Diego F. Gomez-Casati, Mariana Martín, Maria V. Busi

**Affiliations:** Centro de Estudios Fotosintéticos y Bioquímicos (CEFOBI-CONICET), Universidad Nacional de Rosario, Rosario, Santa Fe, Argentina; Russian Academy of Medical Sciences, RUSSIAN FEDERATION

## Abstract

*Ostreococcus tauri*, the smallest free-living (non-symbiotic) eukaryote yet described, is a unicellular green alga of the Prasinophyceae family. It has a very simple cellular organization and presents a unique starch granule and chloroplast. However, its starch metabolism exhibits a complexity comparable to higher plants, with multiple enzyme forms for each metabolic reaction. Glucan phosphatases, a family of enzymes functionally conserved in animals and plants, are essential for normal starch or glycogen degradation in plants and mammals, respectively. Despite the importance of *O*. *tauri* microalgae in evolution, there is no information available concerning the enzymes involved in reversible phosphorylation of starch. Here, we report the molecular cloning and heterologous expression of the gene coding for a dual specific phosphatase from *O*. *tauri* (OsttaDSP), homologous to *Arabidopsis thaliana* LSF2. The recombinant enzyme was purified to electrophoretic homogeneity to characterize its oligomeric and kinetic properties accurately. OsttaDSP is a homodimer of 54.5 kDa that binds and dephosphorylates amylopectin. Also, we also determined that residue C162 is involved in catalysis and possibly also in structural stability of the enzyme. Our results could contribute to better understand the role of glucan phosphatases in the metabolism of starch in green algae.

## Introduction

The native starch granule appears to be a poor substrate for most amylolytic enzymes and one of the crucial steps in initiating starch degradation is its phosphorylation [1]. Plant starch phosphorylation date since more than 120 years ago and starch phosphate content depend on the plant origin: i.e. cereal starch contains 30-fold less phosphate than tuber starch [2, 3]. The phosphorylation process is carried out by the glucan water dikinase (GWD, which phosphorylates the C6-position of glucosyl residues) and phosphoglucan water dikinase (PWD, which phosphorylates the C3 position) enzymes [4–8]. The addition of phosphate groups to the polymer triggers a perturbation of the granule surface transforming the polysaccharide in a better substrate for the attack of exoamylolytic enzymes. However, the phosphate groups can also obstruct some glucan hydrolytic enzymes. Thus, it has recently become clear that dephosphorylation of glucans is also essential for normal storage polysaccharide degradation in plants as well as in mammals [9–13].

Phosphatases are enzymes that remove phosphate groups from a wide variety of substrates that includes proteins, glucans, nucleic acids and lipids. It has been reported that the *Arabidopsis thaliana* phosphoglucan phosphatases starch excess 4 (SEX4) and like-SEX4 2 (LSF2) are fundamental components in the remobilization of leaf starch at night since they dephosphorylate glucans to provide access to amylases to release maltose and glucose [10, 13–16]. The absence of these phosphatases disrupts starch breakdown, resulting in plants that accumulate starch in excess [15–17]. The most outstanding example of the importance of glucan phosphatases in human biology is the Lafora disease, an autosomal recessive disorder which causes neuronal deterioration with accumulation of insoluble, intracellular, hyperphosphorylated carbohydrates called Lafora bodies [18, 19]. Lafora disease is caused by mutations in either the gene encoding Laforin (a glucan phosphatase) or Malin (an E3 ubiquitin ligase) [12, 18, 19].

Many studies have postulated that glucan phosphatase activity is required to regulate the metabolism of storage carbohydrates or amylopectin-like material across multiple kingdoms [10, 13, 20]. However, there is no information available about the function of these enzymes in the ancestral green algae that evolve into present land plants. Based on this, we decided to explore the presence and function of glucan phosphatases in the picoalgae *Ostreococcus tauri*. *O*. *tauri* is the smallest known free-living eukaryote and has a very simple genome and cellular organization although exhibiting the same degree of complexity as that of vascular plants regards to the starch metabolism pathways [21].

The present work reports the identification of a novel *A*. *thaliana* orthologue of LSF2 present in *O*. *tauri*. We perform the biochemical characterization of the first green algae glucan phosphatase informed to date. *O*. *tauri* dual specific phosphatase, herein named OsttaDSP, was found to be a homodimeric enzyme with a Michaelis Menten-type hyperbolic response to amylopectin dephosphorylation. We also found that the C162 residue is important for its catalytic activity and presumably for conformational stability.

These results could contribute to further understand phosphoglucan phosphatase’s role in starch reversible phosphorylation evolution in the green lineage.

## Materials and methods

### *Ostreococcus tauri* culture conditions

*Ostreococcus tauri* strain (OTTH 0595-genome), was obtained from the Roscoff Culture Collection and cultivated in filtered sterile sea water (S9883, Sigma-Aldrich, St. Louis, MO, USA) supplemented with Keller enrichment medium (NCMA, Bigelow Laboratory for Ocean Sciences, USA). The culture was grown under constant gentle agitation at 20°C and subjected to light/dark (12 h/12 h) cycles (50 μE cm^-2^). Protein extraction was performed according to TRIzol Reagent (Thermo Fisher Scientific, Waltham, MA, USA) protein isolation procedure.

### Bioinformatics analysis

#### Database searches and sequence analysis

Protein and nucleotide similarity searches were carried out using the NCBI BLAST server (https://blast.ncbi.nlm.nih.gov/Blast.cgi [22]). Conserve domain searching was done using the NCBI CD-search tool (https://www.ncbi.nlm.nih.gov/Structure/cdd/wrpsb.cgi). Sequence alignments were generated using CLUSTALW [23].

Subcellular localization prediction was carried out with PredAlgo program [24].

#### Homology modeling of OsttaDSP

3D structural models were obtained using the protein structure homology server SWISS-MODEL using OsttaDSP mature sequence as query. Models were evaluated with the ProSA-web structure analysis program [25, 26] and Verify-3D [27, 28]. Protein structural visualization and Fig preparation were conducted using PyMOL (The PyMOL Molecular Graphics System, Version 1.3, Schrödinger, New York, USA).

### Cloning, expression, and purification of OsttaDSP

The coding sequence of OsttaDSP corresponding to the mature OsttaDSP protein (lacking the first 26 amino acid residues that comprise the predicted chloroplast transit peptide) was synthesized, sequenced and cloned into plasmid pUC57 (Genscript, Piscataway, NJ, USA). This vector was digested with BamHI and HindIII to yield a 745 bp fragment. The product was purified and subcloned into the BamHI and HindIII sites of a pRSFDuet-1expression vector (Novagen EMD Biosciences Inc, Madison, WI, USA). The resulting recombinant plasmid pDUET-OsttaDSPΔcTP (PJCDSP01), containing the sequence coding OsttaDSP lacking the chloroplast transit peptide (cTP) with an N-terminal His_6_ tag, was transformed into *Escherichia coli* BL21(DE3) pLys cells and the strain was grown overnight at 37°C in Luria-Bertani (LB) medium supplemented with 30 μg/ml kanamycin. The overnight cultures were inoculated (1:100) into 500 ml of fresh LB medium with the same antibiotic and cultured at 37°C until the optical density at 600 nm reached 0.4–0.5. Isopropyl β-D-thiogalactopyranoside (IPTG) was then added to a final concentration of 0.5 mM, followed by induction for 3 hours at 37°C. The cells were collected by centrifugation, washed, and resuspended in lysis buffer (20 ml of 50 mM Tris-HCl (pH 7.5), 250 mM NaCl, 20 mM imidazole, 1 mM DTT, 1 mM PMSF) and disrupted by sonication. The debris was removed by centrifugation at 12,000 x *g* for 20 minutes at 4°C. The filtered supernatant was loaded onto a 1.0 ml HiTrap Chelating HP column (GE Healthcare bio-Sciences, Uppsala, Sweden). After washing with 50 mM Tris-HCl (pH 7.5), 250 mM NaCl, 20 mM imidazole, recombinant OsttaDSP was eluted using a linear gradient from 20 to 500 mM imidazole in the same buffer. Recombinant protein eluted in the fraction corresponding to 160 mM imidazole. The purified protein was desalted to eliminate imidazole using an Amicon Ultra-10K concentrator (Millipore Corp, Bedford, MA, USA) using a buffer containing 50 mM Tris-HCl pH 7.5, 100 mM NaCl, 1 mM DTT and 1 mM PMSF, and the concentrated protein was stored at -80°C with the addition of 15% (v/v) glycerol until use. Protein samples were analyzed using denaturing SDS-PAGE with 12% gels. Gel filtration chromatography was performed using an AKTA Purifier with a HiPrep 16/60 & 26/60 Sephacryl S-300 High-Resolution size exclusion column (GE Healthcare bio-Sciences), calibrated with a Molecular Weight Calibration Kit (MWGF1000, Sigma-Aldrich).

### Protein quantification

The protein amounts were quantified by Lowry using BSA as the reference standard [29].

### HPLC-MS protein analysis

A sample of purified OsttaDSP protein was run under denaturing conditions on polyacrylamide 12% (w/v) gels and visualized by colloidal Coomassie blue G-250 staining. The band was excised from the gel, transferred to 1.5 ml microcentrifuge tube and sent to the facilities provided by the CEBIQUIEM (Facultad de Ciencias Exactas y Naturales, Universidad de Buenos Aires, Argentina) for analyses. The sample was reduced (20 mM DTT, 45 min, 56°C), alkylated (20 mM iodoacetamide, 45 min, in the dark at room temperature) and digested overnight with trypsin (12 h, 37°C). The digested mixture was lyophilized, resuspended in 10 μl formic acid 0.1% (w/v) and analyzed by nano-high performance liquid chromatography coupled with electrospray ionization mass spectrometry (nano-HPLC-ESI MS, Thermo Scientific technology). The collected data was analyzed with Proteome Discoverer^™^ software (version 1.4).

### Site-directed mutagenesis

Site-directed mutagenesis was performed using the QuikChange II XL Site-Directed Mutagenesis Kit (Agilent Technologies, Santa Clara, CA, USA) according to the manufacturer’s protocol. The following oligonucleotide was used to obtain the recombinant OsttaDSP variant with the predicted catalytic cysteine residue replaced by serine (Cys162Ser): 5´CCATACCGGCGGTAGAGTGCAGGTAGACG-3´.

### Phosphatase activity determination

#### *p*NPP dephosphorylation

Phosphatase activity determinations were carried out for both enzymes in a plate using *p*NPP (N-3254, Sigma-Aldrich) as a substrate as described [30]. Briefly, the assays were performed in 50 μL containing 1X phosphatase buffer (100 mM sodium acetate, 50 mM Bis-Tris, 50 mM Tris-HCl (pH 7), 2 mM DTT and *p*NPP. Following the addition of the phosphatase (300 ng), assays were incubated at 37°C for the specified time followed by the addition of 200 μL of 0.25 M NaOH to stop the reactions; then, absorbance was measured at 410 nm.

#### Amylopectin dephosphorylation

Activity against solubilized amylopectin from potato starch (10118, Sigma-Aldrich) was determined for OsttaDSP and OsttaDSPC162S by measuring released orthophosphate using the malachite green reagent as described [31]. The assays were performed in 20 μL containing 1X phosphatase buffer (100 mM sodium acetate, 50 mM Bis-Tris, 50 mM Tris-HCl (pH 7.5), 2 mM DTT) and amylopectin. Following the addition of the phosphatase (300 ng), assays were incubated at 37°C for the specified time. The reaction was stopped by the addition of 20 μl of 0.1 M N-ethylmaleimide and 80 μl of malachite green reagent. Absorbance was measured after 30 min at 620 nm and the pmoles of phosphate released/min per μg of protein were calculated from using the values calculated from a standard curve.

Assays were corrected for non-enzymatic amylopectin dephosphorylation or amylopectin contamination with free phosphate quantiying the amount of phosphate at each amylopectin concentration. Phosphate contamination of enzyme preparations was negligible (0.07 pmol PO_4_/ ng enzyme).

### In-gel protein phosphatase assay

Sample preparation and native PAGE was performed according to Bollag and Edelstein [32]. All protein samples were prepared in 65 mM Tris-HCl (pH 6.8) containing 10% (v/v) glycerol and 0.01% (w/v) bromophenol blue and then were loaded on gels pre-run 2 h at 4°C (30 min at 100 V, 30 min at 150 V and 1 h at 200 V). Once the protein samples were loaded to gels, native PAGE was performed at a constant voltage of 200 V at 4°C using a 8% separating gel (8% (w/v) acrylamide– 0.22% (w/v) bisacrylamide, 250 mM Tris-HCl, pH 8.8, 6 x 8 cm x 1 mm) with an electrophoresis buffer containing 25 mM Tris-HCl and 192 mM glycine (pH 8.8).

Following native PAGE, the gels were either stained with Coomassie Blue or developed for phosphatase activity using *p*NPP according to Carrillo et al. [33]. The electrophoresed gels were directly soaked in 5 ml of reaction mixture containing 50 mM Bis-Tris-HCl (pH 8.0), 2 mM DTT and 25 mM *p*NPP. Gels were incubated at 37°C until the yellow bands began to appear [33]. The stained gels were scanned at different times as the reaction proceeded.

### Polysaccharide-binding assays

#### Affinity gel electrophoresis

Protein (5 μg) in sample buffer (65 mM Tris-HCl, pH 6.8, containing 10% (v/v) glycerol and 0.01% (w/v) bromophenol blue) was subjected to native 8% (w/v) PAGE at pH 8.8 containing 0.3 mg/ml (or more) amylopectin from potato starch (10118, Sigma-Aldrich) [34]. BSA protein served as reference. Control gels without the addition of polysaccharide were run simultaneously, and proteins were stained with Coomassie blue stain. Amylopectin was prepared according to Wilkens et al 2016 [34].

#### Cosedimentation assay

Purified proteins above described (20 μg each) were mixed with amylopectin from potato (10118 Sigma-Aldrich) in 20 mM Tris–HCl (pH 7.5), at a final polysaccharide concentrations of 10% (w/v). Controls were performed without polysaccharide. Binding was done at 20°C by orbital mixing for 30 min. Polysaccharides were obtained by centrifugation at 12000 × *g* for 5 min, and the supernatant removed and boiled in SDS loading buffer as described [35].

The pellets were washed three times with 100 μL of 20 mM Tris–HCl, pH 7.5 by gentle vortexing and centrifugation, and then boiled in SDS loading buffer in a final volume equal to the supernatants for SDS-PAGE [14].

Protein levels were determined by densitometry from SDS-PAGE gels after staining with 0.25% Coomassie Blue R-250 in 45% (v/v) methanol, 10% (v/v) acetic acid, then destained in 25% (v/v) methanol, 7% (v/v) acetic acid, using a scanner and Gel-Pro Analyzer (Version 4.0). BSA was used as negative control [36].

#### Circular dichroism (CD)

Far-UV CD spectra were obtained using a Jasco J-810 spectropolarimeter (Jasco Inc., Easton, MD, USA) over the wavelength range from 195 to 250 nm, at 25°C. Measurements were performed in a 1 cm quartz cuvette at a rate of 100 nm/min, bandwidth of 1 nm, response time of 2 s, data pitch of 1 nm, and accumulation of 10 as described [37]. CD data are shown as the mean residue ellipticity (deg cm^2^/dmol) obtained after subtracting the baseline, smoothing, and data normalization. CD spectra for OsttaDSP (0.1–0.5 mg/ml) were recorded in 20 mM K-phosphate buffer, pH 7.4.

#### Thermal stability assay

Determination of the melting temperature (*Tm*) values was performed by thermally- induced incorporation of SYPRO Orange (Thermo Fisher Scientific Inc, S6651) into the unfolding protein with analysis using a Q-PCR thermal cycler (Appied Biosystems StepOne^™^). The assays were performed in 20 μL in a MicroAmp^™^ Fast Optical 48-Well Reaction Plate (Applied Biosystems) containing 5X SYPRO Orange solution, 1X protein purification buffer (50 mM Tris-HCl pH 7.5, 100 mM NaCl, 1 mM DTT, 1 mM PMSF and 15% (v/v) glycerol) and 5.4 μM test protein (OsttaDSP or OsttaDSP C162S). Controls contained SYPRO Orange in buffer. All the assays were performed in duplicated and protein samples were centrifuged (10 min, 10000 rpm, 4°C) before being aliquot to the plate. Then the plates were sealed using MicroAmp^™^ 48-Well Optical Adhesive Film (Applied Biosystems) and heated from 25°C to 95°C in the Q-PCR thermal cycler with a ramp speed of 0.5°C per min. The unfolding of the protein was monitored by the increase in fluorescence of the fluorophore. Fluorescence intensities were plotted against temperature for each sample well and transition curves were fitted with the Boltzmann equation using GraphPad software (version 6.01). The midpoint of each transition curve was calculated to estimate the *Tm* of each protein.

### Immunoblot analysis

Immunological detection by immunoblotting was done according to the method of Bollag and Edelstein [32]. Samples were subjected to SDS-PAGE as previously described and transferred to a 0.45 μm nitrocellulose membrane (Amersham Biosciences, UK) at a constant current of 100 mA for 1 h at 4°C. Membranes were then rinsed with TBS, blocked with 3% (w/v) bovine serum albumin ON at 20°C, and washed three times with TBS for 10 min. Blots were then probed with an a-His antibody (Santa Cruz Biotechnology Inc, Dallas, TX, USA) or polyclonal antibodies raised against recombinant *A thaliana* SEX4 [33] affinity-purified against OsttaDSP as described by Plaxton [38]. After primary antibody incubation, membranes were washed three times in TBS for 10 min and then incubated with 1:30000 a-rabbit IgG alkaline phosphatase conjugated or a-rabbit IgG peroxidase conjugate (for *O*. *tauri* crude extracts analysis) secondary antibodies (Sigma-Aldrich) for 1 h at 22°C. Blots were then carefully washed before detection with BCIP-NBT or Amersham ECL Prime detection reagent (GE Healthcare bio-Sciences) as outlined by the manufacturer.

## Results

### Sequence analysis and homology modeling

SEX4, LSF2 and Laforin are classified in the family of atypical Dual-Specificity Phosphatases (DSPs), which belongs to the Protein Tyrosine Phosphatase (PTP) superfamily. To evaluate if this family is also present in green algae, we investigate this fact in the picoalgae *O*. *tauri*. Thus, LSF2 (OAP02459) or SEX4 (OAP03402) protein sequences were used as queries for a tBLASTn search (https://blast.ncbi.nlm.nih.gov/Blast.cgi) against *O*. *tauri* database. *In silico* analysis revealed two loci (Ot02g06340 and Ot13g02940) coding for two dual specificity phosphatases (XP_003075237 and XP_003082765, respectively). Of these two hits founded, Ot02g06340 gave the lowest E-value (7.00 x 10^−62^) when LSF2 was used as query (the E-value for Ot13g02940 was 2.00 x 10^−14^). The alignment of the mature amino acidic sequence of XP_003075237 with mature LSF2 and SEX4 polypeptides showed that XP_003075237 shares a 41% identity and 58% similarity with LSF2 and a 26% identity and 41% similarity with SEX4. On the other hand, the alignment of XP_003082765 with both *A*. *thaliana* glucan phosphatases, indicated that this putative protein shares a 20.4% identity and 34.3% similarity with LSF2 and a 17.3% identity and 30.3% similarity with SEX4. Bioinformatic protein domain prediction tools predicted a DSP domain but not carbohydrate binding domain (CBM) in both XP_003075237 and XP_003082765 putative proteins. To corroborate *in silico* that XP_003075237 and XP_003082765 are LSF2 orthologs, a tBLASTn search in the *O*. *tauri* transcriptome was performed using as query the amino terminus of XP_003075237 or XP_003082765 (lacking the DSP and C-terminal domain) as well as the C-terminal domain of XP_003075237 or XP_003082765 (lacking the DSP and N-terminal domain). We also performed a homology model of the N- and C- terminal regions of XP_003075237 (aa 1–89 and 201–271) or XP_003082765 (aa 1–89 and 201–277) using @atome Platform (http://atome.cbs.cnrs.fr/AT23/index.html). In all cases no protein containing a CBM could be identified. As a consequence, one would infer the presence of two *DSP* genes homologous to *LSF2* in *O*. *tauri* genome. In this work, we decided to continue working with XP_003075237 (Ot02g06340) because of its highest identity to its plant counterpart LSF2.

**Fig 1:**
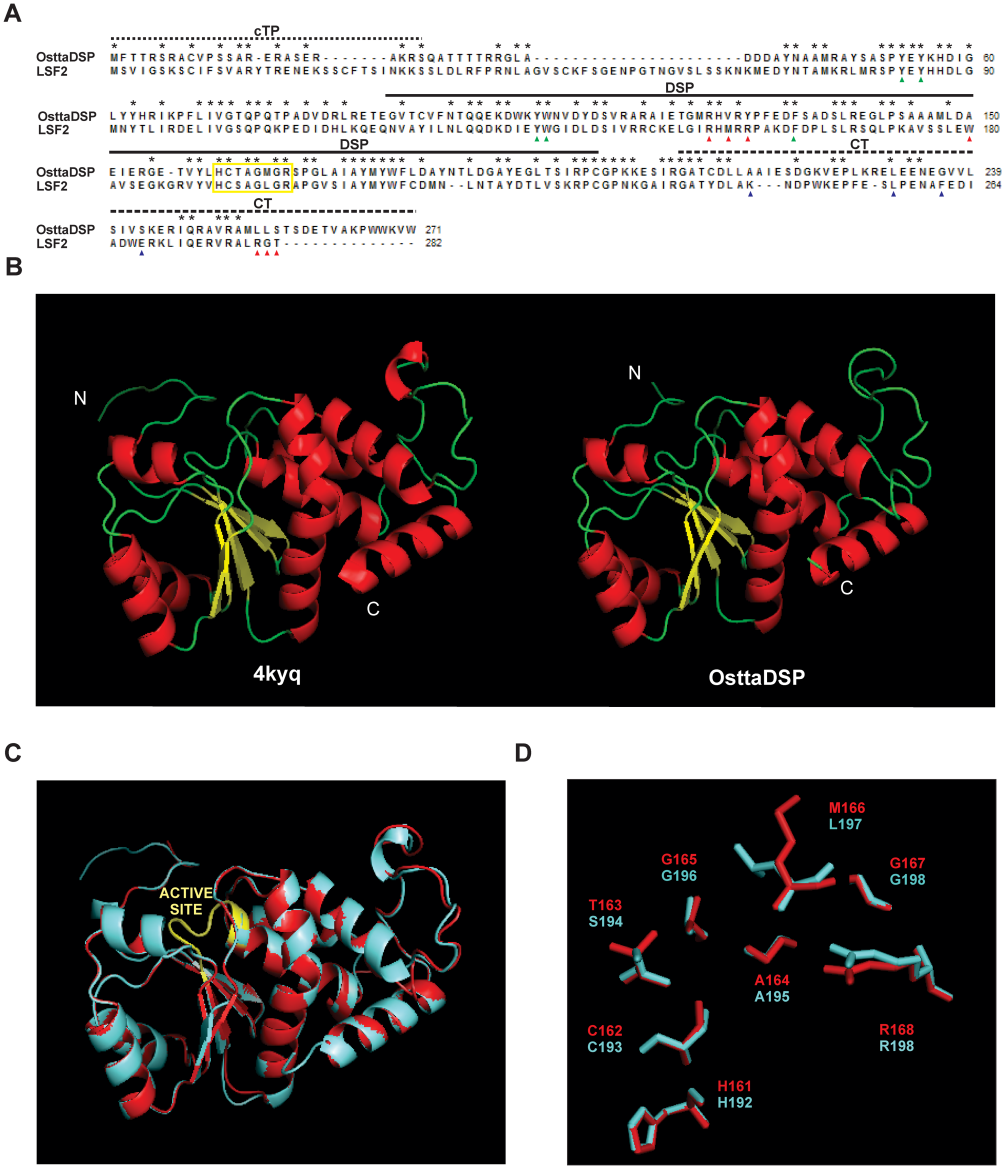
Amino acidic sequence alignment and homology modelling of OsttaDSP. **A)** Amino Acidic Sequence Alignment of OsttaDSP with *A*. *thaliana* LSF2. The yellow box highlight the active site. cTP, chloroplast transit peptide; DSP, dual specific phosphatase and CT, C-terminal. Residues involved in glucan binding in *A*. *thaliana* LSF2 are indicated within the aromatic channel (green arrowheads), SBS2 (red arrowheads) and SBS3 (blue arrowheads). Residues in the aromatic channel are conserved between OsttaDSP and LSF2, however, residues in SBS2 and SBS3 are not. **B)** Structural model of *A*. *thaliana* LSF2 (4kyq, left) and the proposed model for OsttaDSP (right). α- helix are indicated in red, β- sheets in yellow and loops in green. N, N-terminal and C, C-terminal. **C)** Superposition of LSF2 (cyan) and OsttaDSP (red) structures. The active site is shown in yellow. **D)** Superposition between OsttaDSP model and LSF2 structure showing the residues involved in the active site. OsttaDSP residues are shown in red and LSF2 residues are shown in cyan.

The full-length *OsttaDSP* gene (Ot02g06340) contains 816 bp and codes for a putative 271 amino acid polypeptide (pre-OsttaDSP) with an estimated molecular mass of 30.6 kDa. The deduced amino acid sequence of pre-OsttaDSP shares high sequence identity with its counterpart from *A*. *thaliana*: 37.4% identity and 52.3% similarity and the same domain topography (Fig 1A and S1 Fig). Analysis using PredAlgo [24] sowed that pre-OsttaDSP contains a predicted 26 amino acid chloroplastic transit peptide at the N-terminus (Fig 1A), suggesting that OsttaDSP is targeted to the chloroplast and rendering a mature protein of 27.7 kDa.

The primary structure of OsttaDSP catalytic domain has almost all conserved residues that are involved in substrate catalysis as well as substrate binding through the aromatic channel [39–41]. OsttaDSP and LSF2 active sites have all these homolog residues: His161, Cys162, Ala 164, Gly165, Gly167 and Arg168 (OsttaDSP numbering) but Thr163 and Met166 in the algal enzyme are replaced by Ser194 and Leu197 in the plant enzyme (Fig 1A and 1D). All residues forming the aromatic channel in LSF2 are conserved in OsttaDSP: Tyr83, Tyr85, Tyr135, Trp136 and Phe162 (LSF2 numbering) (depicted with green arrowheads in Fig 1A). However, the possible residue determinants in substrate binding of OsttaDSP at the two other surface binding sites (SBSs) are less conserved respect to those in *A*. *thaliana* LSF2 [39]. In reference to SBS2, of the 7 amino acids present in LSF2´s SBS2 (Arg153, Met155, Arg157, Trp180, Arg280, Gly281, and Thr262), only one is conserved in OsttaDSP (Arg153), and two are conservative changes (Arg280 and Thr282) (red arrowheads in Fig 1A). Of the four amino acids conforming SBS3 (Lys245, Leu256, Phe261 and Glu268), only Leu256 is conserved in OsttaDSP (Leu231) (blue arrowheads in Fig 1A).

A homology model of the OsttaDSP protein was built using the 3D structure of *A*. *thaliana* LSF2 glucan phosphatase (PDB code 4kyq, 48.5% identity for residues 51–254) [39]. Analysis using the Verify 3D program showed 90.7% of positive score values, 80% of these higher than 0.2, while a Z-score of –7.07 was obtained using Prosa II. According to these results, we found that the OsttaDSP model is of good quality. The monomeric OsttaDSP model exhibited a fold similar to LSF2 with both α-helical and β-sheet secondary structures conserved (Fig 1B). The alignment of the polypeptide backbone structures of OsttaDSP and LSF2 showed that both 3D structures are very similar (Fig 1C). The alignment was performed using the main chain atoms of residues 51–254 for OsttaDSP and 79–282 for LSF2. Alignment and structural analysis show that the catalytic Cys162 residue, equivalent to Cys193 of LSF2, is well conserved. A closer view of the active site region of both the OsttaDSP model and the template LSF2 is shown in Fig 1D. It is interesting to note that, the amino acid residues of the active site loop (His192, Cys193, Ser194, Ala195, Gly196, Leu197, Gly198, and Arg199, LSF2 numbering) are well conserved, with the exceptions of the Ser194 residue (Thr163 in OsttaDSP) and the Leu197 residue (Met166 in OsttaDSP) (Fig 1D).

### Expression and purification of OsttaDSP

**Fig 2:**
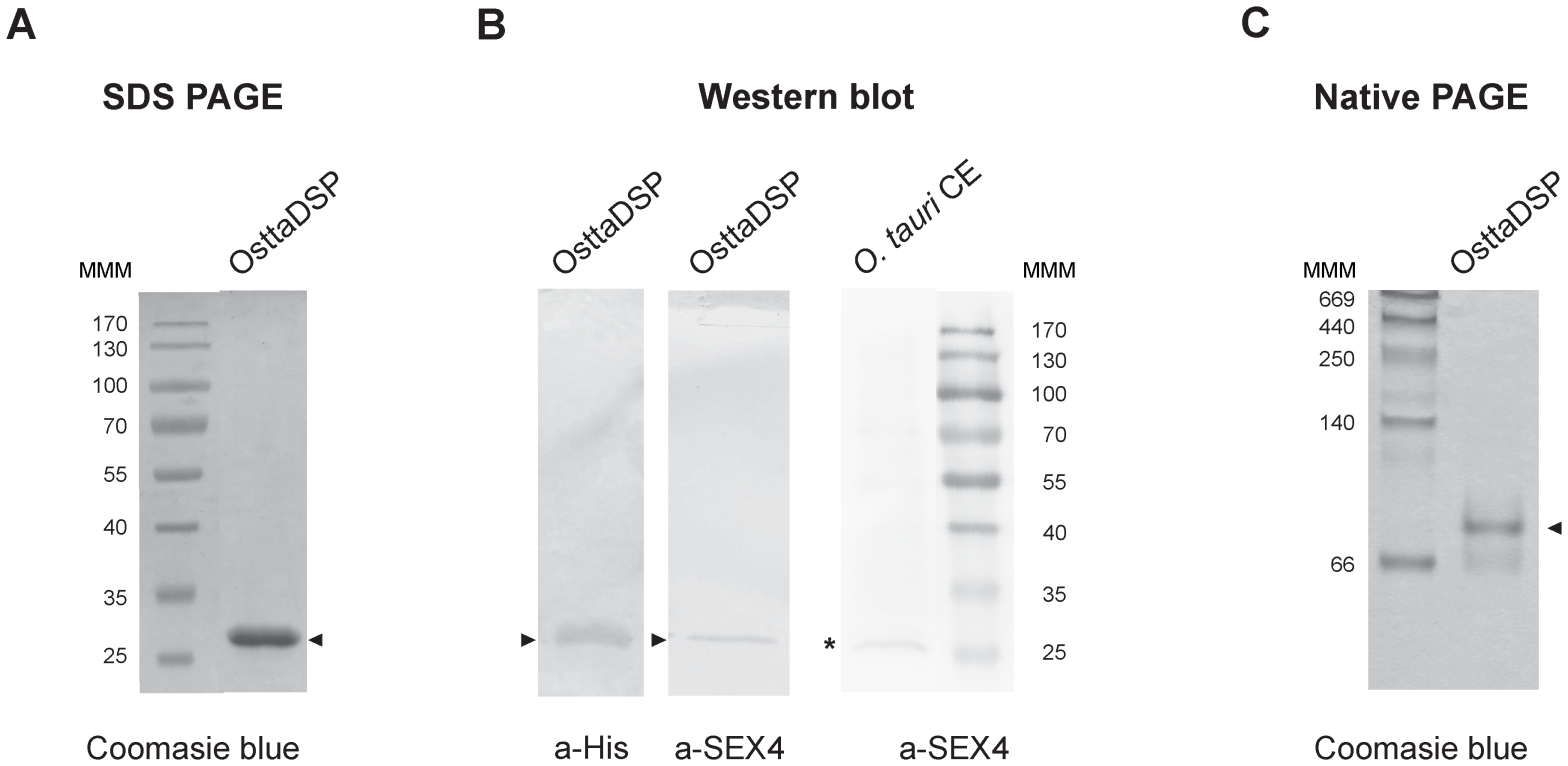
Electrophoretic analysis of OsttaDSP. **A) Coomassie Blue staining of a SDS-PAGE of OsttaDSP. B) SDS-PAGE analyzed by western blotting of OsttaDSP and crude extract of *O*. *tauri* cells.** a-His, a-His antibody; a-SEX4, a-SEX4 antibody immunopurified for OsttaDSP protein. **C) Coomassie Blue staining of a native PAGE of OsttaDSP.** Arrowheads (►) indicate pure recombinant OsttaDSP and asterisk (*) indicate putative DSPs in the crude extract of *O*. *tauri* cells. For SDS-PAGE 1 μg of OsttaDSP was loaded while 3 μg of protein was used for native PAGE analysis. Numbers indicate the molecular masses of markers in kDa. For SDS-PAGE, Page Ruler Prestained Protein Ladder (range 10–170 kDa) was used (Thermo Fisher Scientific, Waltham, MA USA) and, for native electrophoresis, the markers were Amersham High Molecular Weight Calibration Kit (range 66–669 kDa). MMM, molecular mass markers. OsttaDSP, *O*.*tauri* OsttaDSP, *O*. *tauri* CE, *O*. *tauri* crude extract (20 μg).

OsttaDSP was heterologously expressed in *E*. *coli* BL21 pLys strain at 37°C and purified to homogeneity by Ni^2+^ affinity chromatography. The His_6_-tagged protein has a molecular mass of 29.3 kDa and produced a single band at approximately 26 kDa on SDS-PAGE (Fig 2A), which was confirmed by western blot analysis using a-His antibody and affinity-purified a-SEX4 antibody (Fig 2B). We also performed nano-HPLC-ESI MS of the protein to unequivocally confirm the identity of the recombinant protein. All the 21 peptides obtained matches with UniProtKB—Q01DU4 (Q01DU4_OSTTA) with a score of 1024.32 and a 73.53% of coverage.

Size-exclusion chromatography showed a single peak for OsttaDSP (S2 Fig) corresponding to an estimated mass of 54.4 kDa, which is very close to the theoretical size of a homodimer (55.4 kDa), strongly indicating that OsttaDSP is a homodimeric protein in the assayed conditions. Furthermore, OsttaDSP migrates as a single band in native gels as it is shown in Fig 2C.

To analyze the expression of dual specific phosphatases *in vivo*, the affinity-purified a-SEX4 antibodies were used to probe western blots of *O*. *tauri* total protein extracts obtained with TRIzol (Invitrogen, Carlsbad, CA, USA). These blots showed only one specific band of crude extracts of *O*. *tauri* cells with a molecular mass of around 26 kDa. This result suggests that protein/s, recognized with a-SEX4 antibodies inmunopurified with OsttaDSP, is/are present in *O*. *tauri* cells crude extracts (Fig 2B).

### Kinetic characterization of OsttaDSP

OsttaDSP *in vitro* phosphatase activity was assayed towards the small synthetic molecule *para*-nitrophenyl phosphate (*p*NPP) as well as its biologically substrate, amylopectin. In the latter case, phosphate released from amylopectin by OsttaDSP is detected by a colorimetric method due to the formation of a phosphomolybdate malachite green complex [31].

OsttaDSP activity was strictly dependent on the presence of a thiol-reducing compound DTT (dithiothreitol) 2 mM in the assay medium for maximum activity, as previously described for other DSPs [15, 17, 42].

**Fig 3:**
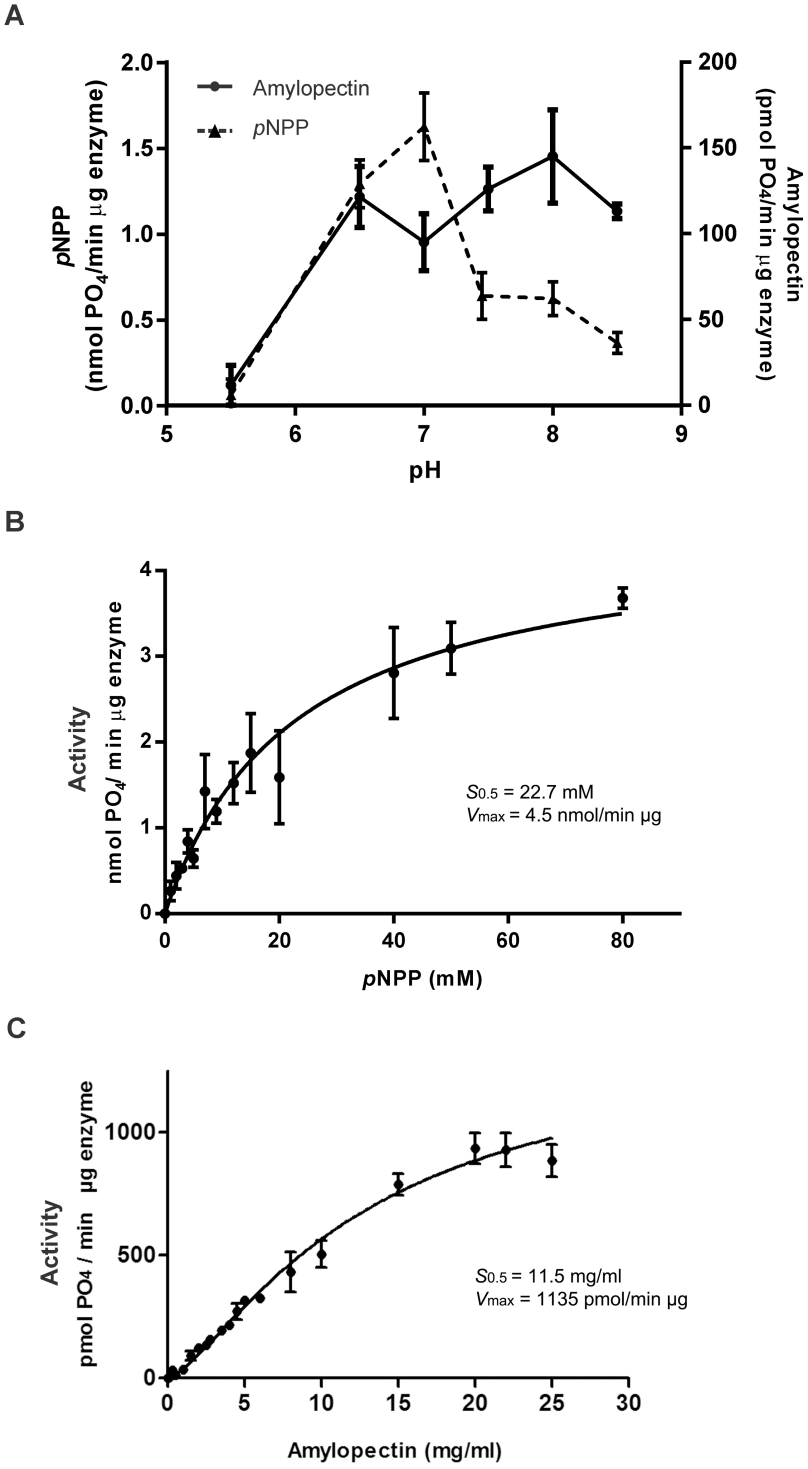
Kinetic characterization of OsttaDSP. **A) Effect of pH on the activity of recombinant OsttaDSP.** Activity was assayed with *p*NPP (filled triangles/▲) or amylopectin (filled circles/●) as a substrate using 100 mM Sodium Acetate, 50 mMBis-Tris and 50 mM Tris as a buffer. Buffer pH values were adjusted to the values shown in the graphic and used to assay the enzyme. All data are the means ± SD of 3 independent experiments. **B) *p*NPP saturation plot for OsttaDSP determined at pH 7. C) Amylopectin saturation plot for OsttaDSP determined at pH 7.5.**

Purified OsttaDSP activity responded to pH in a different manner depending on the substrate being analyzed. The *p*NPP dephosphorylating reaction showed the strongest pH dependence. The rate of amylopectin dephosphorylation displayed a maximum in the range of pH 6.5 to 8.5 (Fig 3A), whereas *p*NPP dephosphorylation showed maximum velocity when assayed at pH 7 (Fig 3A). Thus, we determined the kinetic parameters for *p*NPP and amylopectin dephosphorylation reactions at pH 7 and 7.5, respectively (Fig 3B and 3C).

The saturation plot obtained when the rate of reaction was analyzed as a function of *p*NPP was hyperbolic with an *S*_0.5_ value of 22.7 ± 3.4 mM and a *V*_max_ of 4.5 ± 0.3 (nmol PO_4_/min μg protein) (Fig 3B). The maximum activity assayed with this substrate is around half of that obtained from *A*. *thaliana* LSF2 [15].

Also, the plots of the initial reaction rate of OsttaDSP against amylopectin concentration showed a hyperbolic response (Fig 3C) characterized with an *S*_0.5_ of 11.5 ± 1.2 mg/ml and a maximal velocity of 1135.0 ± 64.3 (pmol PO_4_/min μg protein). Thus, the specific activity obtained is about 5-fold higher respect to that previously informed for the *A*. *thaliana* LSF2 [15].

### Characterization of OsttaDSP C162S modified protein

**Fig 4:**
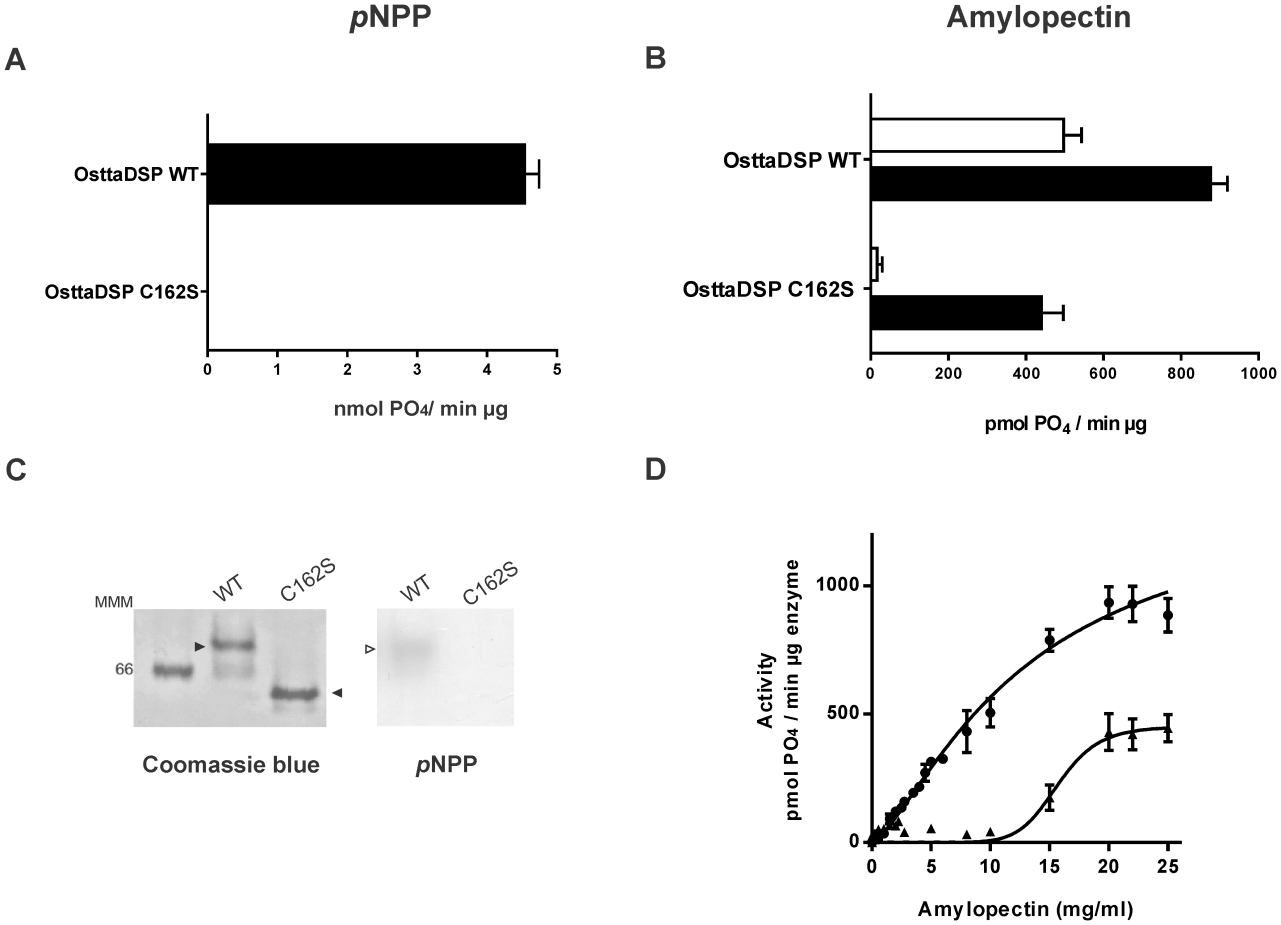
Characterization of OsttaDSP C162S modified protein. **A) Specific activities of OsttaDSP wild-type (WT) and C162S (C162S) enzymes with *p*NPP as substrate.** Both activities were determined at 100 mM of *p*NPP. All data are the means ± SD of 3 independent experiments. **B) Specific activities of OsttaDSP wild-type (WT) and C162S (C162S) modified enzyme in the presence of amylopectin.** Both activities were determined at 10 mg/ml (white bars) or 20 mg/ml (black bars) of amylopectin. All data are the means ± SD of 3 independent experiments. **C) Native PAGE analysis of OsttaDSP wild-type (WT) and C162S mutated enzyme (C162S).** OsttaDSP WT (3 μg) and OsttaDSP C162S (3 μg) were resolved by native PAGE. After native PAGE, gels were either stained with Coomassie Blue or with the activity gel assay using *p*NPP. Filled arrowheads (►) indicate purified recombinant enzymes stained with Coomassie Blue and empty arrowhead (>) indicates OsttaDSP WT stained with the *p*NPP in gel activity assay. The number indicates the molecular mass marker in kDa. MMM, molecular mass marker (Amersham High Molecular Weight Calibration Kit (range 66–669 kDa)). **D) Amylopectin kinetics of OsttaDSP wild-type (WT) and OsttaDSP C162S modified protein (C162S).** Amylopectin saturation plots for OsttaDSP WT (filled circles (●)) and OsttaDSP C162S variant (filled arrowheads (►)) were performed at pH 7.5. All data are the means ± SD of 3 independent experiments.

Glucan phosphatases as being members of PTPs possess an invariant catalytic cysteine at the active site. In accordance with the protein sequence alignment of OsttaDSP with LSF2 (Fig 1), the Cys162 residue should play this catalytic role. Hence, to confirm Cys162 functionality we produced a site-directed mutant for this residue performing a conservative change: the cysteine residue was exchanged to serine. The resulting enzyme, named OsttaDSPC162S, was tested with *p*NPP or amylopectin as substrates (Fig 4). OsttaDSPC162S lacks *p*NPP phosphatase activity but shows some amylopectin phosphatase activity (Fig 4A and 4B). This mutated enzyme showed a specific activity of less than 10 pmol PO_4_/min μg protein and about 400 pmol PO_4_/min μg protein when assayed at 10 mg/ml and 20 mg/ml amylopectin respectively. The pH dependency of the activity of OsttaDSPC162S enzyme was also examined (S3 Fig). OsttaDSP and OsttaDSPC162S showed constant maximum activity in pH range from 6.5 to 8.5 when analyzed at 20 mg/ml amylopectin (S3 Fig). Thus, we then analyzed the response of OsttaDSPC162S mutant protein with increasing amylopectin concentration at pH 7.5 (Fig 4D). OsttaDSPC162S displayed an interesting kinetic behavior when assayed with amylopectin as substrate; plots of the initial reaction rate against amylopectin concentration showed a sigmoidal response (Fig 4D) with a Hill coefficient (*n*_H_) of 4. This reaction was characterized with a *S*_0.5_ of 17.1 ± 1.9 mg/ml and a maximal velocity of 425 ± 51 (pmol PO_4_/min μg protein). The latter result is coincident with the results obtained for LSF2 and SEX4 since a mutation of the active-site Cys to Ser causes the total loss of both enzyme activities when assayed using *p*NPP as well as amylopectin at low concentration (2.25 mg/ml) [15, 40]. No reports are showing that both, SEX4 or LFS2, were tested with amylopectin at high concentrations nor the kinetic properties of enzymes related to starch dephosphorylation in photosynthetic organisms.

When OsttaDSPC162S was analyzed on native PAGE and detected by Coomassie Blue staining, it seemed to behave as a monomer rather than being a dimer as its wild-type counterpart (Fig 4C). In-gel activity assays confirmed the lack of phosphatase activity of OsttaDSPC162S towards *p*NPP (Fig 4C). Thus, OsttaDSPC162S lost *p*NPP phosphatase activity in-gel as well as in solution but maintained some amylopectin phosphatase activity at high amylopectin concentrations with an increase of about 1.5–fold in the *S*_0.5_ for amylopectin (Fig 4D).

**Fig 5:**
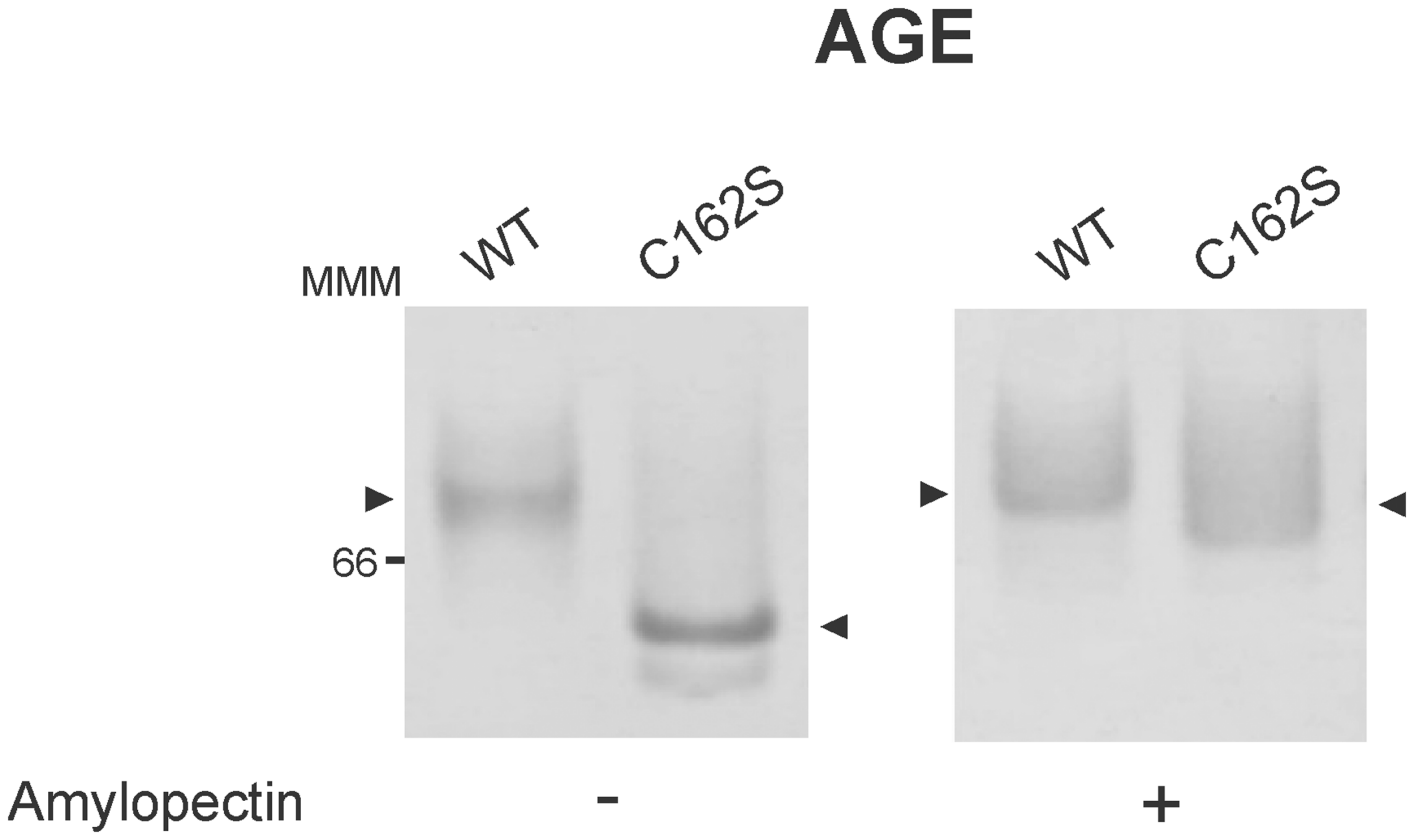
Affinity gel electrophoresis (AGE) of OsttaDSPwild-type (WT) and C162S mutated protein (C162S). OsttaDSP WT (WT, 5 μg) and OsttaDSP C162S (C162S, 5 μg) proteins were run on native PAGE without or with amylopectin (0.3 mg/ml) simultaneously under the same conditions. Gels were then revealed by Coomassie Blue staining. The number indicates the molecular mass marker in kDa. MMM, molecular mass markers (Amersham High Molecular Weight Calibration Kit (range 66–669 kDa)).

Based on this result, it is possible to speculate that the enzyme is active on amylopectin as a monomer or that the presence of its natural substrate somehow induces its oligomerization. To test this, we performed affinity gel electrophoresis (AGE) in the presence of amylopectin (0.3 mg/ml). As it is shown in Fig 5, OsttaDSPC162S migrated faster in native PAGE (as a monomer) but showed a clear retardation on AGE with amylopectin. In the latter case, OsttaDSPC162S migrated in parallel to OsttaDSP wild type leading to hypothesized that the mutant variant dimerizes in the presence of amylopectin. It is worth mentioning that neither OsttaDSP nor OsttaDSPC162S showed a more significant retardation if amylopectin concentration is increased in gels. However, by co-sedimentation assays, we could determine that both enzymes bind amylopectin to the same extent (S4 Fig).

Furthermore, it is important to mention that the mutation C162S did not result in any appreciable conformational change of the secondary structure of the enzyme since the circular dichroism (CD) spectrum of the mutant was very similar to that of the wild-type enzyme (S5 Fig). Also, the thermal stability of wild type and mutant proteins were analyzed by differential scanning fluorometry. Both proteins gave almost the same melting temperature around 39°C.

## Discussion

In agreement with *in silico* studies, the green lineage of glucan kinases and phosphatases seemed to be quite well conserved from green algae to land plants, highlighting the essential nature of reversible glucan phosphorylation [16, 43, 44]. The analysis of *O*. *tauri* genome shows the presence of two *DSP* genes (Ot02g06340 and Ot13g02940) and five genes coding for five putative starch phosphorylating enzymes (Ot13g01510, Ot16g02370, Ot04g04170, Ot08g01260 and Ot08g01280). Up to date, there are no data on the characterization of phosphatases enzymes, nor any explanation about the dikinase multiplicity in this organism. According to the *in silico* analysis, both *DSP* genes codify for LSF2 orthologs suggesting that the DSPs would be highly conserved throughout the green lineage, including the oldest genomes like *O*. *tauri*.

As described previously, of the two *DSP* genes found in *O*. *tauri*, we continue working with Ot02g06340 because of its protein product (XP_003075237) has the highest identity to its plant counterpart LSF2 from *A*. *thaliana*. The full-length *OsttaDSP* gene (Ot02g06340) contains 816 bp and codes for a 245 amino acid mature polypeptide with high sequence identity with LSF2. Like LSF2, OsttaLSF2 lacks a CBM reported up to date, but it efficiently binds and dephosphorylates amylopectin, constituting an active enzyme (Fig 3 and S4 Fig). The structure of *Arabidopsis* LSF2 showed that this protein engages a glucan chain by an aromatic channel and two SBSs nearly 20 A° away from the active site [39, 45]. While the aromatic channel is fully conserved in OsttaDSP (green arrowheads in Fig 1A), this is not the case for SBS2 and SBS3 (Fig 1, red and blue arrowheads). Since SBSs are short stretches of non-contiguous amino acids that come together to impart glucan binding, they have a heterogeneous topology, and there is no a strict SBS signature motif [16]. This is why is difficult to identify OsttaDSP SBSs just via bioinformatics tools.

The biochemical characterization of OsttaDSP showed that it migrates as a single band in SDS-PAGE and native gels (Fig 2). Using gel filtration chromatography, the enzyme was detected as a 54.4 kDa protein (S2 Fig), suggesting that OsttaDSP is a homodimer. The kinetic plot of OsttaDSP in the presence of amylopectin showed a hyperbolic kinetics with an *S*_0.5_ of 11.5 ± 1.2 mg/ml. Is worth mentioning that the kinetic properties of enzymes related to starch dephosphorylation in photosynthetic organisms are not known.

As is known from previous studies, PTPs have the signature motif HCX_5_R, which contains the invariant cysteine residue at the base of the active site cleft [46, 47]. This cysteine exists as an active thiolate anion during catalysis and is stabilized by the electrostatic interactions of the neighboring histidine [47]. The arginine of the signature motif coordinates the cysteinyl-phosphate intermediate that is formed after a nucleophilic attack by the thiolate ion of cysteine. Transfer of the phosphate is promoted through the protonation of the dephosphorylated substrate by the aspartic acid residue located approximately 30 residues upstream the signature motif. Phosphate is released after hydrolysis by the afore mentioned aspartate and a nearby glutamine to restore the thiolate anion. *Arabidopsis thaliana* LSF2 and SEX4 are active using *p*NPP or amylopectin as substrates, and within the DSP active-site, the conserved Cys was reported to be essential for activity [15, 20, 30]. Mutation of the corresponding Cys in LSF2 (Cys193) to Ser abolished its activity against *p*NPP and amylopectin (at 2.25 mg/ml) analogous to the C198S mutation in SEX4 [15, 20, 48]. In this work, the mutated enzyme OsttaDSP C162S retains some activity at higher concentrations of amylopectin, but this activity is lost when pNPP was used as the substrate (Fig 4A and 4B). The single Cys to Ser aminoacidic substitution resulted in the change of hyperbolic kinetics to the sigmoidal one in response to amylopectin concentration (Fig 4D). This explains why the enzyme shows very low activity at subsaturating amylopectin concentrations, but it retains about 40% of the wild type enzyme activity at saturating amounts of amylopectin. In addition, the evidence detected by native electrophoresis showed that the formation of a homodimer might also be lost with the C162S mutation (Fig 5), suggesting an involvement of this residue both, in catalytic activity and structural stability. From the results presented here, we can infer that Cys162 is critical for OsttaDSP activity and that possibly local structural effects resulting from the Cys to Ser mutation renders the mutant enzyme capable of performing the dephosphorylation of amylopectin in the whole range of pH analyzed (Fig 4 and S3 Fig). However, further investigation is necessary to fully understand the implications of this conservative point mutation in the catalytic mechanism of OsttaDSP as well as in the final structure of the active site.

Our results could contribute to a better understanding of starch metabolism in this microalga and provide the basis for a better evolutionary characterization of these dual nature phosphatases. While SEX4 is described to hydrolyze both C6- and C3-bound phosphate, LSF2 is unique as it is highly specific for the C3-position of glucosyl residues of starch, although it also has low capacity to dephosphorylate some C6-esters [15]. The absence of a SEX4 ortholog n *O*. *tauri* raises the possibility that OsttaDSP displays both activities or that the dephosphorylation of C6-phosphorylated residues is carried out by the other DSP found in *Ostreococcus*. The determination of algae glucan phosphatases structures would reveal further insights into their mechanism of activity and specificity in particular because of the presence of SBSs. Specific role(s) of the SBSs are an active area of research because the observed synergistic function could be due to altered dynamics from proximity effects, distinct binding preferences, or global effects on glucan stability. Furthermore, the specific properties regarding the regulation of the polysaccharide metabolism are fundamental due to their potential biotechnological and industrial applications.

## Supporting information

S1 Fig Schematic representation of OsttaDSP domain structure in comparison with *A*. *thaliana* SEX4 and LSF2. OsttaDSP contains a chloroplast-targeting peptide (cTP) at its N-terminus, followed by a dual-specificity phosphatase (DSP) domain and a C-terminal (CT) motif. DSP contains a cTP, a DSP domain and CT motif. SEX4 contains a cTP at its N-terminus, followed by a DSP domain, a carbohydrate binding module (CBM) of the CBM48 family, and a CT motif.
(TIF)

S2 Fig Determination of the oligomeric state of OsttaDSP. The elution profile from a size-exclusion chromatography of affinity-purified mature OsttaDSP show only one peak corresponding to an estimated molecular mass of 54.4 kDa suggesting that OsttaDSP has a dimeric quaternary structure.
(TIF)

S3 Fig Effect of pH on the activity of recombinant OsttaDSP (filled circles/●) and OsttaDSPC162S (filled triangles/▲) enzymes. Activity was assayed with amylopectin (20 mg/ml) as a substrate using 100 mM Sodium Acetate, 50 mM Bis-Tris and 50 mM Tris as a buffer. Buffer pH values were adjusted to the values shown in the graphic and used to assay the enzyme. All data are the means ± SD of 3 independent experiments.
(TIF)

S4 Fig Binding of OsttaDSP and OsttaDSP C162S to potato amylopectin *in vitro*. Both proteins were incubated with (+) or without (-) amylopectin for 30 min at 20°C. The polysaccharide was pelleted by centrifugation. Proteins in the supernatant (S) and bound to the pellet (P) were visualized by SDS-PAGE and Coomassie blue staining.
(TIF)

S5 Fig Far-UV CD spectra of recombinant wild-type OsttaDSP and the mutant C162S. The CD spectra were detected, and the mean residue ellipticity was calculated as described in Materials and Methods.
(TIF)
