# Supplementary figures and images for: Identification and analysis of OsttaDSP, a phosphoglucan phosphatase from Ostreococcus tauri
Source: PLoS One. 2018 Jan 23;13(1):e0191621. doi: 10.1371/journal.pone.0191621 (PMC5779698; doi:10.1371/journal.pone.0191621)

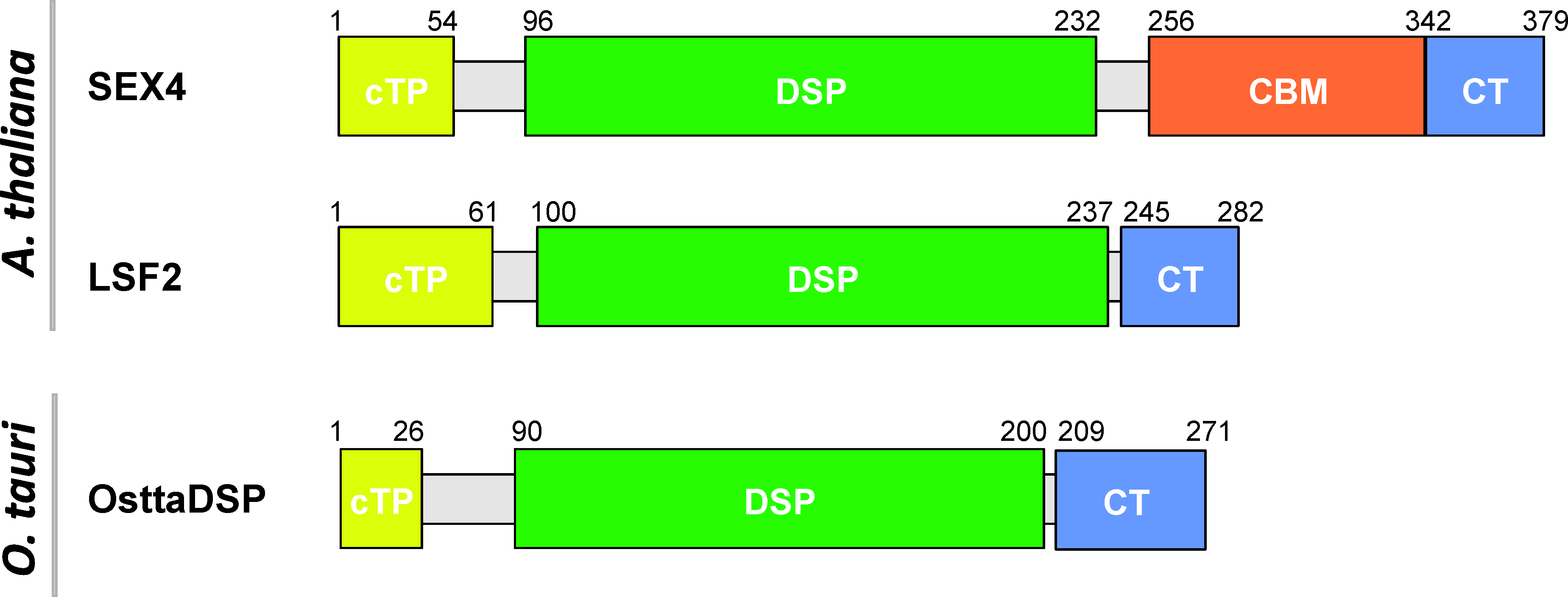

Supplement: S1 Fig — OsttaDSP contains a chloroplast-targeting peptide (cTP) at its N-terminus, followed by a dual-specificity phosphatase (DSP) domain and a C-terminal (CT) motif. DSP contains a cTP, a DSP domain and CT motif. SEX4 contains a cTP at its N-terminus, followed by a DSP domain, a carbohydrate binding module (CBM) of the CBM48 family, and a CT motif. (TIF) [file pone.0191621.s001.tif]

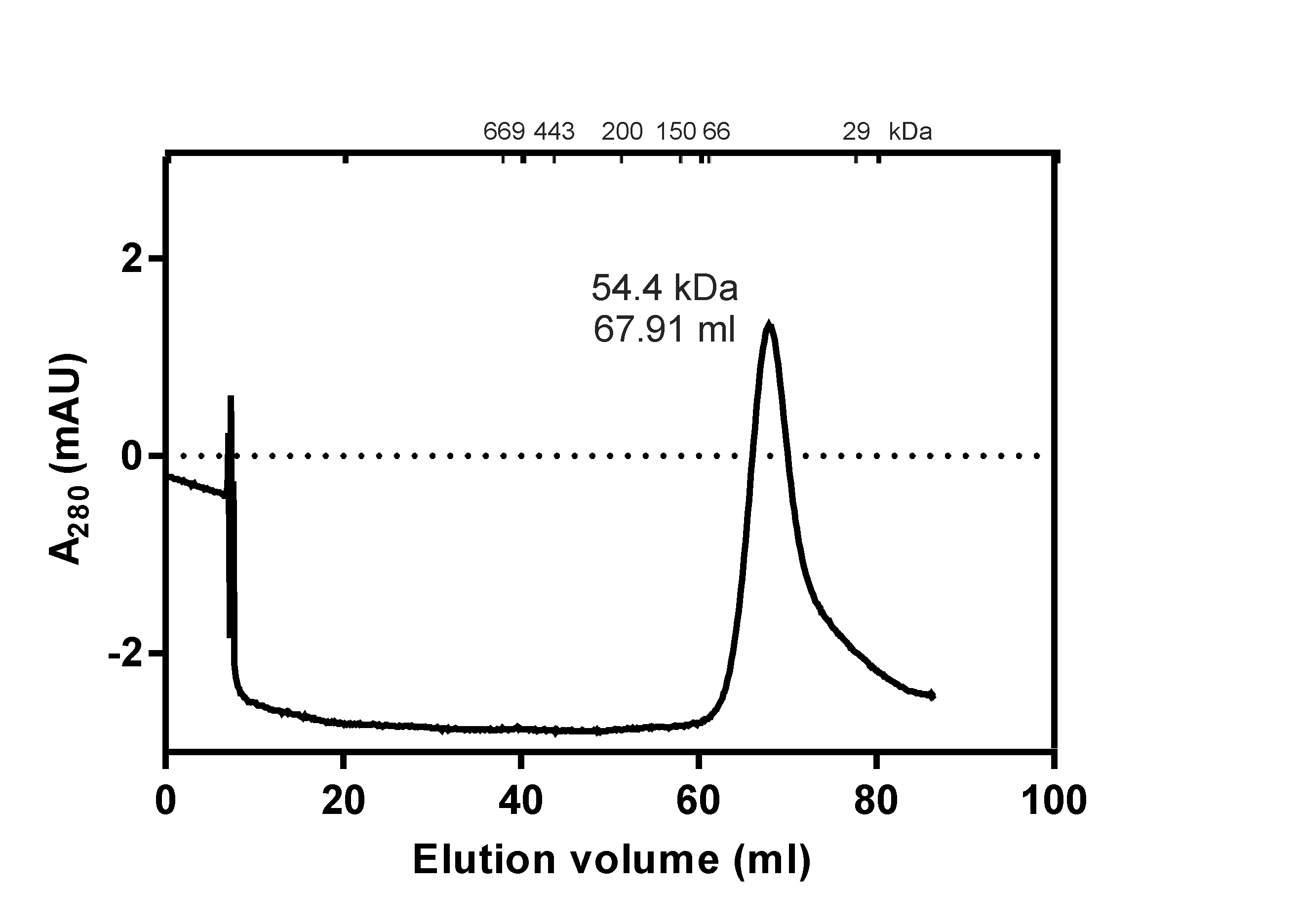

Supplement: S2 Fig — The elution profile from a size-exclusion chromatography of affinity-purified mature OsttaDSP show only one peak corresponding to an estimated molecular mass of 54.4 kDa suggesting that OsttaDSP has a dimeric quaternary structure. (TIF) [file pone.0191621.s002.tif]

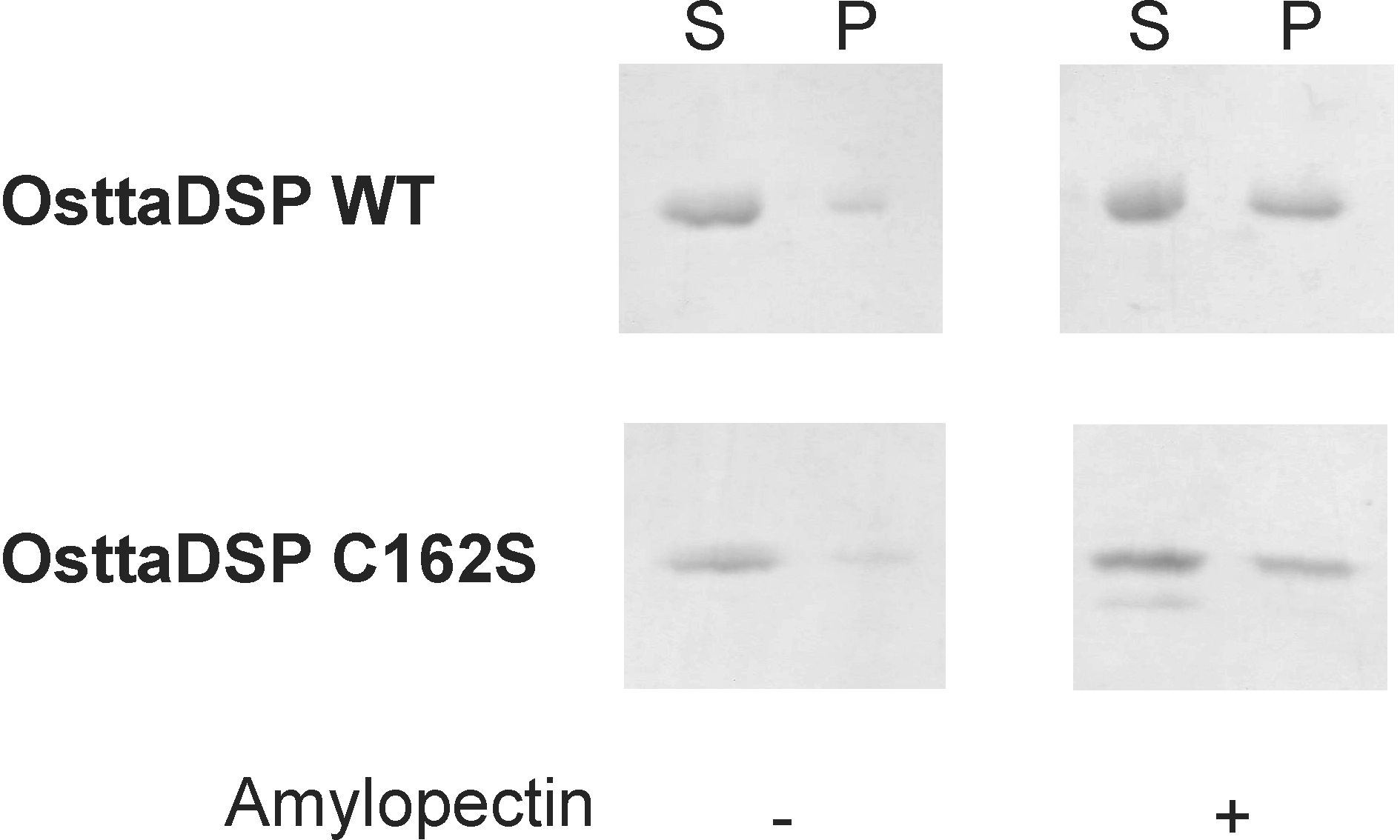

Supplement: S4 Fig — Both proteins were incubated with (+) or without (-) amylopectin for 30 min at 20°C. The polysaccharide was pelleted by centrifugation. Proteins in the supernatant (S) and bound to the pellet (P) were visualized by SDS-PAGE and Coomassie blue staining. (TIF) [file pone.0191621.s004.tif]

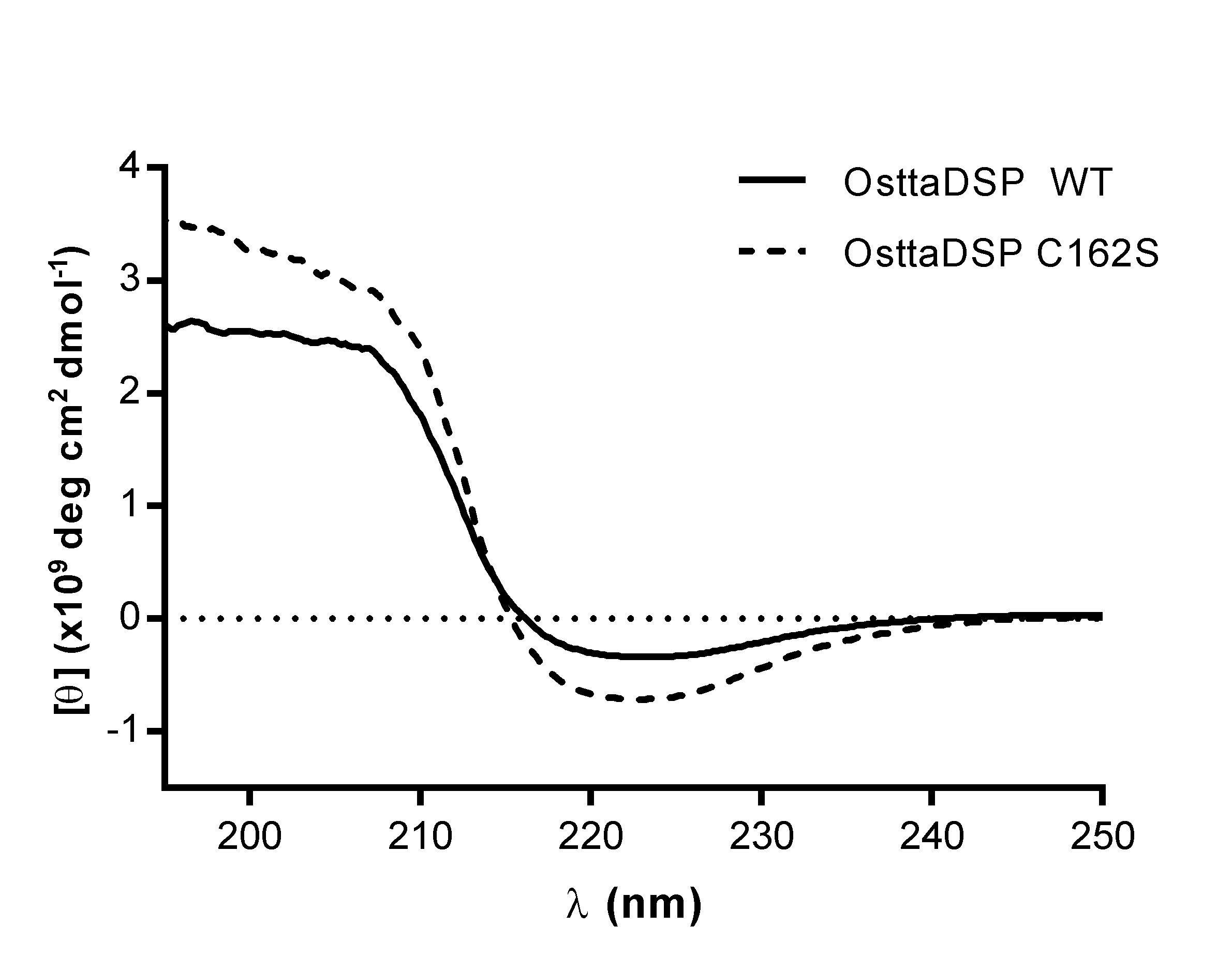

Supplement: S5 Fig — The CD spectra were detected, and the mean residue ellipticity was calculated as described in Materials and Methods. (TIF) [file pone.0191621.s005.tif]
